# Systematic Analysis of Covalent and Allosteric Protein Kinase Inhibitors

**DOI:** 10.3390/molecules28155805

**Published:** 2023-08-01

**Authors:** Elena Xerxa, Oliver Laufkötter, Jürgen Bajorath

**Affiliations:** LIMES Program Unit Chemical Biology and Medicinal Chemistry, Department of Life Science Informatics, B-IT, Rheinische Friedrich-Wilhelms-Universität, Friedrich-Hirzebruch-Allee 5/6, D-53115 Bonn, Germany

**Keywords:** protein kinases, covalent inhibitors, allosteric inhibitors, warheads, potency, promiscuity, X-ray structures, binding sites

## Abstract

In drug discovery, protein kinase inhibitors (PKIs) are intensely investigated as drug candidates in different therapeutic areas. While ATP site-directed, non-covalent PKIs have long been a focal point in protein kinase (PK) drug discovery, in recent years, there has been increasing interest in allosteric PKIs (APKIs), which are expected to have high kinase selectivity. In addition, as compounds acting by covalent mechanisms experience a renaissance in drug discovery, there is also increasing interest in covalent PKIs (CPKIs). There are various reasons for this increasing interest such as the anticipated high potency, prolonged residence times compared to non-competitive PKIs, and other favorable pharmacokinetic properties. Due to the popularity of PKIs for therapeutic intervention, large numbers of PKIs and large volumes of activity data have accumulated in the public domain, providing a basis for large-scale computational analysis. We have systematically searched for CPKIs containing different reactive groups (warheads) and investigated their potency and promiscuity (multi-PK activity) on the basis of carefully curated activity data. For seven different warheads, sufficiently large numbers of CPKIs were available for detailed follow-up analysis. For only three warheads, the median potency of corresponding CPKIs was significantly higher than of non-covalent PKIs. However, for CKPIs with five of seven warheads, there was a significant increase in the median potency of at least 100-fold compared to PKI analogues without warheads. However, in the analysis of multi-PK activity, there was no general increase in the promiscuity of CPKIs compared to non-covalent PKIs. In addition, we have identified 29 new APKIs in X-ray structures of PK-PKI complexes. Among structurally characterized APKIs, 13 covalent APKIs in complexes with five PKs are currently available, enabling structure-based investigation of PK inhibition by covalent-allosteric mechanisms.

## 1. Introduction

Protein kinases (PKs) play a pivotal role in many aspects of cellular physiology [1,2], due to their extensive involvement in many cellular signal transduction processes. Signaling functions of PKs are exerted through adenosine triphosphate (ATP) cofactor-dependent phosphorylation of downstream PKs or other proteins involved in signaling cascades. This activity resides in the catalytic domain of PKs and is controlled through their regulatory domain. Membrane receptor-associated PKs play a pivotal role in trans-membrane signal transduction into the cell, and many other PKs then transmit signals intracellularly through phosphorylation cascades. Considering their ubiquitous and critical role in signaling, abnormal activation of PKs and aberrant or out-of-control phosphorylation activity cause many different pathologies. Accordingly, inhibition of the catalytic activity of PKs using PK inhibitors (PKIs) is the major approach for therapeutic intervention of uncontrolled PK functions. As a consequence, PKIs are among the most intensely investigated drug candidates. Currently, 72 PKIs are approved by the US Food and Drug Administration [3] for the treatment of several types of cancer [1,2,4,5] as well as inflammatory or metabolic diseases [1,2,6,7]. Depending on the therapeutic area, either selective or multi-PK inhibition is desirable. Multi-target activity of compounds is also referred to as promiscuity. For example, for cancer treatment, promiscuous PKIs are often preferred because they can simultaneously interfere with different cancer-related signaling pathways [4]. On the other hand, for the treatment of chronic inflammatory or metabolic diseases, selective PKIs are typically required, also to limit off-target side effects during long-term treatment [6]. Hence, finding a balance between PK selectivity and multi-PK activity, depending on the therapeutic applications, continues to be a critically important issue in PKI drug development. Therefore, different mechanisms of PK inhibition have been extensively explored at the molecular level of detail, for example, through enzyme kinetic analysis or X-ray crystallography. 

Based on experimentally determined binding modes, PKIs are classified into different types [1,8] that are implicated in preferential broad-spectrum or selective PK inhibition. Most PKIs bind to the ATP-binding pocket or proximal to it (type I, I1/2, or II). Considering the conservation of this site across the human kinome, multi-PK activity of ATP site-directed (type I) inhibitors is expected, but this is not generally the case [9,10]. Indeed, a variety of selective ATP site-directed PKIs have been reported [11,12]. In addition, allosteric PKIs (APKIs) binding proximal to (type III) or distant from (type IV) the ATP-binding pocket often display high selectivity [8,13,14]. Other PKIs include bivalent compounds (type V), which target the ATP site and another site in the regulatory or catalytic kinase domain [14,15]. 

While most PKIs act non-covalently, there are also covalent PKIs (type VI) targeting the side chains of different amino acids. In drug discovery, active compounds acting by covalent mechanisms have long been de-prioritized. However, over the past decade, increasing attention has been paid to covalent PKIs (CPKIs, type VI) and other covalent drug candidates because of their frequently observed high potency, prolonged residence times, and the selectivity of covalent inhibitors, as well as improved pharmacokinetics [16,17,18]. CPKIs mostly target free cysteines, but also lysine, serine, tyrosine, threonine, histidine, aspartate, glutamate, tryptophan, methionine, arginine, or proline residues [16,17,18]. Covalent modification of little-considered residues directly or indirectly implicated in ligand binding often yields selective inhibitors. Some CPKIs act through allosteric effects. However, in the absence of structural data, such effects are often difficult to discern. As a prerequisite for inhibition, covalent bond formation is facilitated by nucleophilic attack of electrophilic moieties, commonly termed as “warheads”, targeting a nucleophilic residue. The resulting durable target engagement and often limited spatial constraints for covalent inhibition further extend PK druggability [19]. Although most CPKIs have been discovered serendipitously, drug design using reversible non-covalent ligands as templates [20,21] or fragment-based design [22,23] and tethering approaches [24] are becoming common practice in CPKI discovery and development. Various warheads with different chemical properties are used for CPKIs, likely leading to different binding and inhibition characteristics, which are typically explored on a case-by-case basis for CPKIs of interest. In a recent systematic analysis of PKIs in public databases, an unexpectedly large number of nearly 14,000 CPKI candidates was identified that contained at least one of 13 commonly used warheads and were active against a total of 369 human kinases [9]. Acrylamide and heterocyclic urea were the most frequently occurring warheads, and CPKIs containing these two moieties were further analyzed. They were found to have a generally higher potency than structural analogues without warheads. In addition, it was found that CPKIs with acrylamide, but not heterocyclic urea, were enriched among most promiscuous PKIs [9].

In addition to CPKIs, APKIs are of particularly high interest in PK drug discovery [1,8,12,13]. APKIs are generally expected to have high selectivity because they mostly bind to sites that are only little-conserved across the kinome and elicit inhibitory effects through induced conformational changes, which might represent unique mechanisms of action, depending on the kinase target. While allosteric effects lead to non-competitive inhibition, which can be confirmed through enzyme kinetics, a detailed elucidation and ultimate confirmation of APKI modes-of-action (including allosteric consequences of covalent inhibition) generally depends on the availability of structural data. In 2020, we conducted a systematic analysis of X-ray structures of PK-PKI complexes to identify APKIs and their binding sites [25]. A total of 232 APKIs were discovered that bound to 12 different binding sites distributed across the catalytic domain of different kinases [25]. In some instances, the comparison of apo- and holo-forms of PKs uncovered allosteric mechanisms, including long-range conformation changes; in others, allosteric effects could not be rationalized at the structural level.

In the present work, we further expand the analysis of CPKIs and APKIs. CPKIs containing different types of warheads that are less frequent than acrylamide and heterocyclic urea were investigated, focusing on their potency and promiscuity, both at the global level and in the context of analogue series. Furthermore, we continued our systematic survey of PK-PKI complex structures first carried out three years ago in the search for new structurally confirmed APKIs. Taken together, these studies were conducted to further increase the knowledgebase for kinase drug discovery, focusing on two types of PKIs that currently receive particular attention.

## 2. Results and Discussion

### 2.1. Covalent Protein Kinase Inhibitors

#### 2.1.1. Warhead Distribution

**Figure 1:**
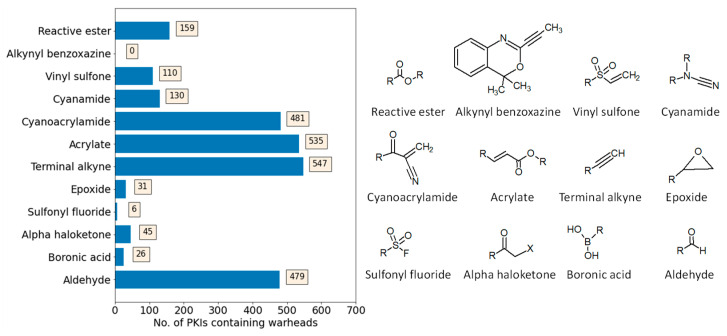
Warhead distribution in covalent kinase inhibitors. The bar chart on the left shows the distribution of 12 warheads in human CPKIs. On the right, warhead structures are depicted.

In a recent large-scale analysis, we identified 155,579 unique PKIs in public databases that were active against 440 human kinases on the basis of curated high-confidence activity data [9]. Rather unexpectedly, these PKIs were found to include 13,949 potential CPKIs that contained 13 commonly used warheads (the presence of a warhead does not necessarily lead to covalent PK inhibition). The warhead distribution was strongly dominated by acrylamide and heterocyclic urea, which were detected in ~87% of all CPKIs [9]. These CPKIs were then further investigated. In this work, we further extend the initial warhead analysis by focusing on CPKIs with 12 other commonly used warheads [19,20,21], including aldehyde, alpha haloketone, acrylate, alkynyl benzoate, boronic acid, cyanoacrylamide, cyanamide, epoxide, reactive ester, sulfonyl fluoride, terminal alkyne, and vinyl sulfone. To systematically identify CPKIs containing these warheads, substructure searches for warhead fragments were conducted in our curated PKI collection (see Section 3). Figure 1 shows the results. For 11 of 12 warheads (except alkynyl benzoxaxine), varying numbers of CPKIs were detected. Cyanoacrylamide, acrylate, terminal alkyne, and aldehyde were most frequently observed: each of these warheads was present in close to or more than 500 CPKIs. In addition, reactive ester, vinyl sulfone, and cyanamide were each found in more than 100 CPKIs. For the remaining 4 warheads (epoxide, sulfonyl fluoride, alpha haloketone, and boronic acid), only limited numbers of CPKIs (6 to 45) were identified, which prohibited a sound statistical assessment of the compound properties. Hence, further analysis was carried out with the CPKIs containing seven different warheads (CPKI subsets), for which >100 to >500 compounds were available. Thus, together with the initial analysis, CPKIs with nine different warheads could be characterized in detail, focusing on their potency and promiscuity compared to non-covalent PKIs. 

#### 2.1.2. Global Potency Analysis

**Figure 2:**
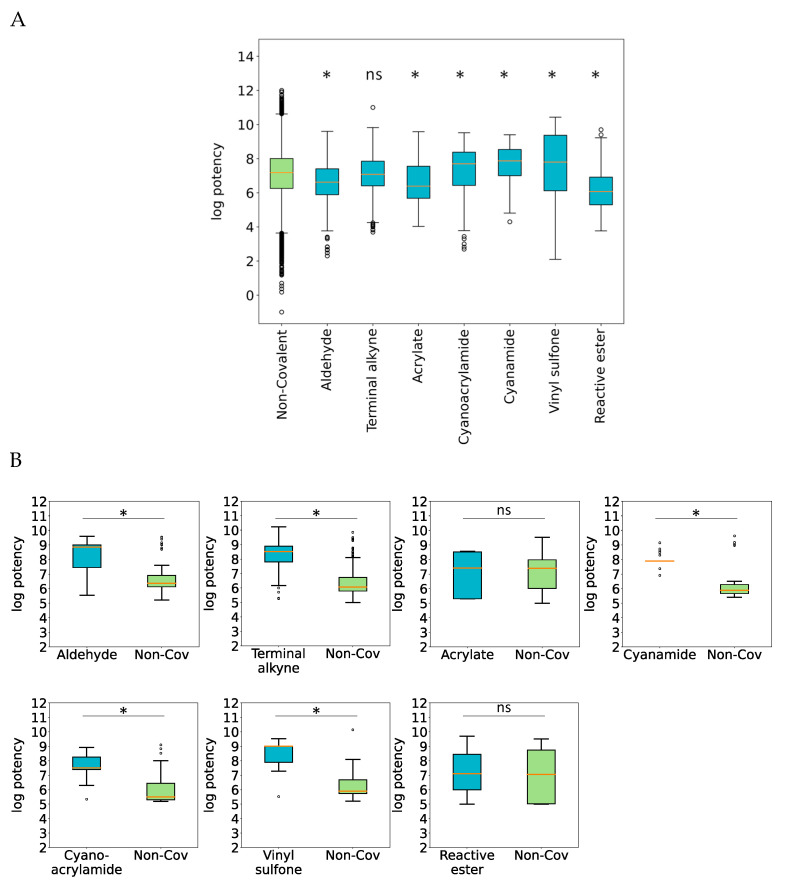
Potency comparison. In (**A**), boxplots show the logarithmic potency distribution for non-covalent PKIs and seven CPKI subsets. Stars indicate statistically significant differences between the PKI distribution and each CPKI distribution, and “ns” means “not significant”. In (**B**), boxplots compare logarithmic potency distributions for CPKIs from each subset and corresponding structurally analogous PKIs. Stars indicate statistically significant differences between corresponding distributions (and “ns” means “not significant”).

One of the issues frequently discussed in the context of CKPI analysis and development is whether or not covalent inhibition might cause a general increase in potency compared to non-covalent modes of PK inhibition. Notably, the presence of warheads potentially leading to covalent PK modification is not the only factor determining inhibitor potency. For example, CPKIs must also contain scaffolds that are complementary to targeted binding sites both in terms of shape and chemical interactions, and they must present warheads in a well-defined manner to access side chains of residues implicated in PK functions and avoid steric hindrance. Furthermore, CPKIs must be stable, despite the presence of reactive groups. Moreover, concerning compound flexibility and hydrophobicity and the resulting entropic effects during binding, the same criteria apply to both CPKIs and PKIs. Hence, the activity of CPKIs cannot be solely attributed to warheads and covalent binding. However, to address the question of whether CPKIs might have a general potency advantage over non-covalent PKIs, potency distributions of large samples of CKPIs and PKIs can be compared, which provides an unbiased general assessment. Therefore, for CPKIs containing each of the seven warheads, the potency distribution was determined and compared to the global potency distribution of non-covalent PKIs. Figure 2A shows the results. Statistical assessment using the one-way ANOVA test indicated a significant difference (*p*-value < 0.0001) between the potency distributions of non-covalent PKIs and CPKIs. Subsequent pairwise comparison via the post-hoc Tukey test confirmed statistically significant differences between non-covalent PKIs and CPKIs containing six of the seven warheads (except terminal alkyne). However, the median potency of only three of these six CPKI subsets (cyanoacrylamide, cyanamide, and vinyl sulfone) was higher (in the mid- to low-nanomolar range) than the median potency of the other PKIs. Thus, on the basis of global potency value distributions, there was no general increase in the potency of CPKIs over other PKIs. Therefore, we next concentrated on the analysis of structural analogues from different CPKI subsets and corresponding non-covalent PKIs.

#### 2.1.3. Potency Differences between Covalent and Non-Covalent Inhibitor Analogues

The comparison of global compound potency distributions does not take PK targets into account. Hence, to complement and further extend global potency analysis, we also carried out a target-centric analysis of potency differences between structurally analogous CPKIs and PKIs. Moreover, structural analogues share the same scaffold, which also represent an important factor for compound potency, as discussed above. Therefore, direct comparisons of CPKIs and PKIs that are structural analogues and active against the same PK target provide more detailed information concerning potential potency differences between CPKIs and corresponding non-covalent PKIs than global potency analysis. Therefore, we systematically identified analogue series consisting of CPKIs and non-covalent PKIs (see Section 3). From each series, PKI–CPKI pairs (structural analogues) with an at least 100-fold difference in potency against the same PK target were extracted. For CPKIs with each of the seven warheads, 62, 161, 57, 34, 31, 72, and 40 qualifying pairs were extracted for the aldehyde, terminal alkyne, acrylate, cyanamide, cyanoacrylamide, vinyl sulfone, and activated ester warhead, respectively. Figure 2B compares the potency of CPKIs with different warheads and corresponding PKIs from analogue pairs. For five of seven CPKI subsets (except acrylate and activated ester warheads), CPKIs had a statistically significant gain in potency over their PKI counterparts of at least two orders of magnitude (*p*-value < 0.00001). Thus, in most cases, CPKIs were the highly potent compound in CPKI–PKI analogue pairs, indicating that CPKIs had a significantly higher potency against a given PK target than structurally analogous PKIs. Thus, local analysis of CPKI–PKI analogues provided a more differentiated picture than global potency analysis and confirmed increases in potency for several CPKI subsets compared to related non-covalent PKIs.

#### 2.1.4. Promiscuity Analysis

**Figure 3:**
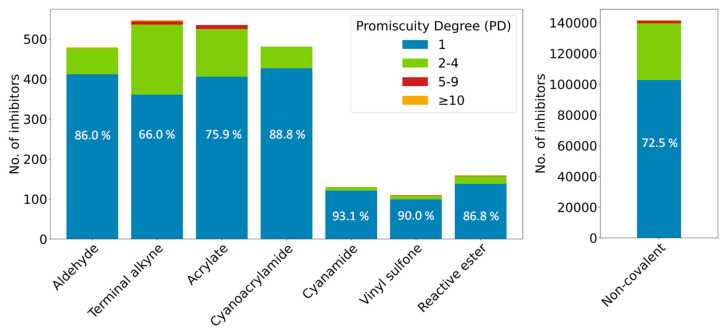
Comparison of promiscuity degrees for covalent and non-covalent kinase inhibitors. For each CPKI subgroup and all non-covalent PKIs, proportions of non-promiscuous (PD 1) and increasingly promiscuous compounds (PD [2–4], [5–9], ≥10) are reported.

In addition to CKPIs’ potency and efficacy, their potential promiscuity is a highly relevant issue for PK drug discovery, especially considering the large number of currently available potential CPKIs [9]. An open question is whether or not CPKIs are more promiscuous than PKIs. Currently, no detailed investigations addressing this question are available for CPKIs with warheads other than acrylamide and heterocyclic urea. Considering that many PKs contain multiple residues that are, in principle, amenable to covalent modification, one might hypothesize an increased likelihood of covalent over non-covalent inhibition, especially if candidate residues such as free cysteines occur in or proximal to ligand-binding regions. As shown previously, CPKIs with acrylamide, but not heterocyclic urea, warheads were enriched among most promiscuous public PKIs [9]. However, from these observations, no firm conclusions could be drawn. Accordingly, we have extended the analysis to CPKIs with different warheads. Notably, promiscuity analysis is generally affected by data incompleteness because most PKIs have not been exhaustively tested against the human kinome (and are only annotated with one or two PKs). Nonetheless, direct comparisons could be carried out for the seven CPKI subgroups and non-covalent PKIs. Figure 3 reports the proportions of different promiscuity degrees (PDs, that is, numbers of PK targets CPKIs/PKIs are reported to be active against) for the seven CPKI subgroups and non-covalent PKIs. Compared to PKIs (72.5% are annotated with a single PK, corresponding to PD 1), only one CPKI subgroup (terminal alkyne) contained a smaller proportion of inhibitors with single-PK activity (66.0% PD 1) and a correspondingly larger proportion of promiscuous inhibitors. In all other cases, the proportion of CPKIs with reported single-PK activity was larger than that for PKIs. The results might partly be influenced by the much smaller sample sizes of CPKIs compared to PKIs. However, based on the results obtained for nine CPKI subgroups (including acrylamide and heterocyclic urea from our initial investigation), a general increase in promiscuity of CPKIs compared to non-covalent PKIs cannot be supported on the basis of currently available activity data. For the further exploration and application of CPKIs for selective PK inhibition, this represents an important finding.

### 2.2. Allosteric Protein Kinase Inhibitors 

We systematically searched for X-ray structures of complexes of human PKs with inhibitors or activators deposited since 2021 in the Protein Data Bank (PDB) [26] and investigated these complexes for the presence of allosteric ligands (see Section 3). All X-ray structures were individually assessed to ensure a high level of confidence of the analysis. 

#### 2.2.1. New Structurally Characterized Inhibitors

A total of 262 PK ligands acting by allosteric mechanisms were identified. Compared to our initial survey conducted three years ago [25], 30 new ligands were identified, including 29 APKIs and one PK activator, as reported in Table 1, which classifies PK ligands by type. Thus, in recent years, there was moderate growth in the number of structurally characterized APKIs. Newly identified APKIs included 11 and 10 type III and IV inhibitors, respectively, three type VI inhibitors, and five multi-site ligands binding to the ATP site and an allosteric site or multiple allosteric sites in different PKs.

#### 2.2.2. Distribution over Allosteric Binding Sites

**Figure 4:**
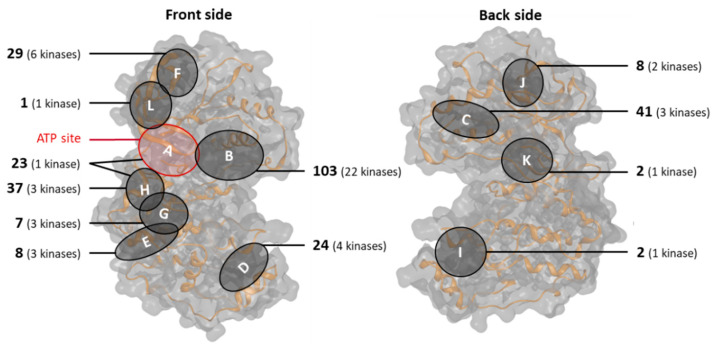
Binding sites of allosteric protein kinase ligands. Shown is the distribution of APKIs, including multi-site ligands and PK activators across 11 allosteric bindings sites mapped on a representative catalytic PK domain.

The binding sites of new PK ligands were mapped. All newly identified compounds bound to five of the 12 previously characterized sites [25]. New allosteric binding sites in the catalytic PK domain were not discovered. Figure 4 shows the distribution of all 262 allosteric ligands (including multi-site ligands) over the 12 binding sites mapped on a representative PK catalytic domain (PDB ID 4EHV). The number of PKs in which each binding site was occupied with crystallographic ligand(s) is also reported. Multi-site ligands were counted multiple times for each binding site in which they were found.

Newly identified allosteric PK ligands bound to the B, E, F, G, and H sites, all of which are located on the front side of the catalytic domain in Figure 4. The single newly identified PK activator was accommodated in the F pocket. Overall, most APKIs were type III inhibitors, binding to the B pocket proximal to the ATP-binding site. Here, 13 new APKIs were identified, some of which were active against three new PKs for which no crystallographic APKIs were previously available. In addition, eight new APKIs bound to the H and four others to the F site, leading to the engagement of two new PKs for each of these sites. Furthermore, for each of the E and G sites, one new APKI was identified, interacting with a previously unobserved PK. Hence, new APKIs further increased the number of human PKs inhibited by allosteric mechanisms.

#### 2.2.3. Covalent Allosteric Inhibitors

Currently, a total of 13 structurally characterized covalent APKIs (type VI) are available, as reported in Table 2 (in the following, PKs and PK groups [27] are named using standard abbreviations [27,28]). All covalent APKIs targeted cysteine residues, and 10 of 13 contained an acrylamide warhead. The covalent APKIs were available in complexes with five PKs. Seven APKIs bound to AKT1, and all of them were located in the B site of the protein. Two APKIs bound to MAPK14 (D site) and two others to CDK2 (I site). The two remaining APKIs complexed PDPK1 (C site) and AURKA (K site) and contained unique warheads (a sulfhydryl and a thiazoline group, respectively). Although most of these covalent APKIs contain acrylamide warheads, they are unlikely to have high promiscuity degrees, considering their allosteric mode of action, which typically restricts the number of PK targets. While structural information concerning covalent APKIs is currently limited, these complexes can be used as a starting point to explore covalent allosteric mechanisms of PK inhibition for five allosteric binding sites.

#### 2.2.4. Kinome Distribution

**Figure 5:**
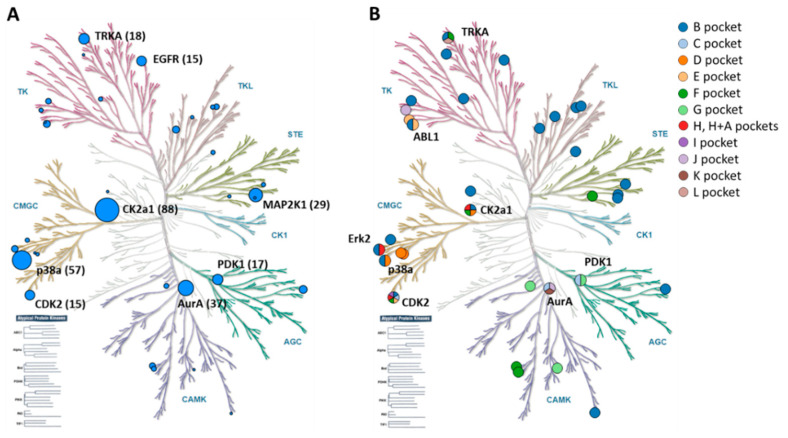
Distribution of allosteric protein kinase ligands across the human kinome. PKs with available ligands were mapped on a phylogenetic tree representation of the kinome and represented as dots. For each PK target, the number of available ligands is provided in parentheses. In (**A**), dots are scaled in size according to the number of APKIs. In (**B**), dots are color-coded according to the number of different allosteric binding sites of APKIs. The most prominent PK targets are named using standard abbreviations.

The kinome distribution of all 262 allosteric PK ligands was also determined, as depicted in Figure 5. PK targets of APKIs were broadly distributed across the human kinome. However, as shown in Figure 5A, most APKIs were available for two Ser/Thr PKs, CK2a1 and p38a (PK group CMGC), followed by AurA (group CAMK) and MAP2K1 (group STE). For Tyr PKs, only comparably few APKIs were detected. Furthermore, as shown in Figure 5B, Ser/Thr PKs also displayed the largest variety of occupied allosteric binding sites, notably CDK2 with 15 APKIs binding to six different allosteric sites and CK2a1 with 88 APKIs binding to four different sites. Thus, in light of the kinome distribution of structurally characterized APKIs and their PK targets, Ser/Thr PKs (group CMGC) represented a hot spot for allosteric PK inhibition.

## *3.* Materials and Methods

### 3.1. Covalent Protein Kinase Inhibitors 

#### 3.1.1. Protein Kinase Inhibitor Data Retrieval and Curation

For PKIs, activity data were extracted from the ChEMBL [29] (version 31) and BindingDB [30] databases (accessed on 3 October 2022) and subjected to systematic curation criteria by applying different confidence criteria. For human PKs, UniProt IDs were retrieved from UniProtKB/Swiss-Prot [28] (release 3 August 2022) and used to query ChEMBL and BindingDB. Compounds with reported activity against these kinases were required to be tested in direct binding assays (relationship type “D”) with standard potency measurements (K_i_, K_d_, or IC_50_) lower than or equal to 10,000 nM and a standard activity relationship “=”. In addition, only assays for single kinases were considered. For ChEMBL, additional curation criteria included the selection of assays with the highest level of confidence (assay confidence score of 9) and the detection of activity records with comments such as “uncertain”, “potential transcription error”, “outside typical range”, or inconsistent “active”/“inactive” tags. Compounds with such activity records were removed. To harmonize data obtained from different databases, compounds were represented as simplified molecular-input line-entry system (SMILES) [31] strings and subjected to a process of canonicalization, neutralization, and removal of salt and stereochemical information. Hence, the resulting standardized SMILES provided unique compound representations for data integration. When multiple qualifying potency measurements of the same type were available for the same inhibitor of a given PK, they were averaged, and the PKI was only retained if the standard deviation was lower than 1 (logarithmic unit). If different standard measurements were available for a PKI, K_i_ or K_d_ values were prioritized over IC_50_ values. 

#### 3.1.2. Identification of Covalent Inhibitors

Potential CPKIs were identified by substructure searching in our curated PKI collection for the presence of 12 commonly used warheads [19] that were encoded as SMILES arbitrary target specification (SMARTS) patterns [32]. Acrylamide- and heterocyclic urea-containing PKIs that were previously studied [9] were not analyzed herein. 

#### 3.1.3. Global Potency Analysis

Potency measurements were recorded as negative decadic logarithmic values and average values were calculated for CPKIs containing the same warhead. Averages were compared via the one-way ANOVA statistical test and for post hoc validation, the Tukey test was applied [33].

#### 3.1.4. Analogue Series, Analogue Pairs, and Promiscuity Degrees

Analogue series consist of compounds with a common core structure and different substituents at one or multiple sites. For the identification of analogues formed by PKIs and CPKIs, the compound–core relationship (CCR) method was used, which systematically partitions compounds into core structures and substituents according to retrosynthetic rules and organizes them into different series [34]. Pairs of analogues from the same series with an at least 100-fold difference in potency against the same PK were identified. Statistical significance was assessed via the paired *t*-test. In addition, for each CPKI/PKI, the PD was calculated as the number of PKs it was active against.

### 3.2. Allosteric Protein Kinase Inhibitors

UniProt IDs of human PKs were used to search for PK entries in the PDB archive deposited since the beginning of 2021. Only PK structures with a resolution of less than 3.5 Å were considered, and PKs with atypical or unrecognizable folds were omitted. Qualifying entries were systematically parsed to detect the presence of a ligand (files containing no ligand or large peptides or peptide derivatives were omitted). Each ligand-containing PK entry was scanned for the presence of any of the keywords: “allosteric”, “activator”, “covalent”, “inhibit”, “noncompetitive”, and “uncompetitive”, and entries containing keywords were retained. Notably, APKIs might act non-covalently or covalently. Each of these PDB structures was visually inspected using the Molecular Operating Environment (MOE) [35] to search for APKIs (or allosteric activators), map their binding sites, and assign them to PKI types. Structural representations were generated with MOE.

## 4. Conclusions

PKs continue to be among the most intensely investigated pharmaceutical targets in a variety of therapeutic areas. In kinase drug discovery, covalent and allosteric PKIs are currently of high interest. In this work, we have investigated CPKIs with different warheads and structurally characterized APKIs on the basis of a systematic analysis of curated activity data (CPKIs) and X-ray structures (APKIs). For seven different warheads, including cyanoacrylamide, acrylate, terminal alkyne, aldehyde reactive ester, cyanamide, and vinyl sulfone, sufficient numbers of CPKIs were identified to enable a statistically sound follow-up analysis. Compared to non-covalent PKIs, statistically significant differences in potency distributions of CPKIs were detected, and some—but not all—CPKIs subsets were overall more potent than non-covalent PKIs. This global comparison implicitly took potency-determining effects into account and was essentially unbiased, but also influenced by the diversity of PKIs and the large number of covered PK targets. Therefore, a subsequent systematic analysis of potency differences captured by structurally analogous CPKI–PKI pairs active against the same PK was carried for different warheads. By definition, these compounds shared the same scaffolds, making corresponding contributions to potency against the same target such that the warhead was the major distinguishing feature. The comparisons confirmed the higher potency of CPKIs relative to the corresponding non-covalent PKIs for most warhead subsets. On the other hand, general increases in promiscuity of CPKIs compared to PKIs could not be detected on the basis of the currently available data. Since data-driven promiscuity analysis is generally affected by data incompleteness, further experimental kinome profiling of CPKIs versus corresponding non-covalent PKIs (such as structural analogues) should provide further insights into the potentially different PK activity profiles. In addition to CPKIs, we have investigated APKIs, which are also preferentially considered for selective PK inhibition. In this case, our focal point has been on X-ray structures because they provide immediate access to allosteric binding sites and potential mechanisms. Most likely, however, only a fraction of the current APKIs have been studied at the structural level thus far (moreover, allosteric effects elicited by some PKIs might not even be known). Therefore, structure-based analysis can currently not (yet) be expected to provide a comprehensive view of allosteric PK inhibition. Through systematic analysis of X-ray structures, we identified a total of 262 allosteric PK ligands, including 242 APKIs (and 20 PK activators), yielding an increase of 29 structurally characterized APKIs (plus 1 activator) over the past 3 years. Hence, there was only moderate growth in the number of newly characterized APKIs, and new allosteric binding sites were not uncovered. However, the kinome distribution of APKIs from X-ray structures and their binding sites revealed Ser/Thr PKs as a major target of allosteric inhibition, rather than Tyr PKs, for which by far the most PKIs are available. Notably, we found that the currently available APKIs included 13 covalent compounds that bound to five different PK targets. Albeit still limited in their number, these compounds and their complex structures provide an initial basis for the structure-based assessment of covalent allosteric mechanisms of PK inhibition, which is another attractive topic for future research. As part of our study, all CPKIs and APKIs we have identified were made freely available with associated target and mechanistic information. These data further expand the knowledgebase for PK drug discovery and enable follow-up investigations on selected CPKIs, APKIs, and PK targets.

## Figures and Tables

**Table 1:** Allosteric kinase inhibitors and activators from X-ray structures.

Ligand Type	Binding Site Description	Count
Type III	Adjacent to ATP site	92 (81)
Type IV	Distant from ATP site	91 (81)
Type V	ATP site + allosteric site	15 (15)
Type VI	Allosteric site	13 (10)
Activators	Allosteric site	20 (19)
Multi-site ligands	ATP site and/or allosteric site(s)	31 (26)
Total		262 (232)

PK ligands from X-ray structures are classified according to PKI and ligand types. Numbers in parentheses report counts from our initial survey.

**Table 2:** Covalent allosteric kinase inhibitors from X-ray structures.

PDB ID	Binding Site	Kinase	Modified Residue	Warhead
6HHJ	B	AKT1	Cys310	Acrylamide
6HHF	B	AKT1	Cys296	Acrylamide
6HHI	B	AKT1	Cys296	Acrylamide
6HHH	B	AKT1	Cys296	Acrylamide
6HHG	B	AKT1	Cys310	Acrylamide
6S9X	B	AKT1	Cys310	Acrylamide
6S9W	B	AKT1	Cys310	Acrylamide
3ORX	C	PDPK1	Cys148	Sulfhydryl
5O8V	D	MAPK14	Cys251	Acrylamide
5O8U	D	MAPK14	Cys252	α,β-unsaturated Carbonyl
5OO0	I	CDK2	Cys177	Acrylamide
5OSJ	I	CDK2	Cys117	Acrylamide
5ORL	K	AURKA	Cys247	Thiazoline

Reported are the X-ray structures (PDB ID) containing covalent APKIs, their warheads, PK targets, covalently modified PK residues, and binding sites.

## Data Availability

CPKIs and APKIs reported herein are freely available via the following links: http://doi.org/10.5281/zenodo.7970944 (CPKIs), http://doi.org/10.5281/zenodo.8144176 (APKIs).
